# Nonlinear interaction between double tearing mode and Kelvin–Helmholtz instability with different shear flows

**DOI:** 10.1038/s41598-023-40920-0

**Published:** 2023-08-21

**Authors:** Z. Li, X. Q. Wang, Y. Xu, H. F. Liu, J. Huang

**Affiliations:** Institute of Fusion Science, School of Physical Science and Technology Southwest, Jiaotong University, Chengdu, 610031 China

**Keywords:** Astrophysical plasmas, Magnetically confined plasmas, Magnetospheric physics, Solar physics, Physics, Applied physics, Astronomy and astrophysics, Fluid dynamics, Plasma physics

## Abstract

The nonlinear interaction between the double tearing mode (DTM) and Kelvin–Helmholtz (KH) instabilities with different shear flow profiles has been numerically investigated via the use of a compressible magnetohydrodynamics (MHD) model. We focus on KH instabilities in weak and reversed magnetic shear plasmas with strong stabilizing effect of field line bending. Results show that KH instabilities coupled with DTMs occur in these plasmas and the KH mode dominates the instability dynamics, suggesting the crucial role of weak magnetic shear in the formation of high-mode harmonics. For symmetric flows, an asymmetric forced magnetic reconnection configuration is maintained during the growth phase, leading to interlocking of the modes. Additionally, this investigation of the DTM-KH instability interaction contributes to our understanding of the nonlinear reconnection mechanism in the regime of weak and reversed magnetic shear plasmas, which is relevant for astrophysical and fusion studies.

## Introduction

Plasma flow-driven instabilities play an important role in magnetized plasmas, including the solar corona, magnetospheric, and astrophysical jets^1–7^. Plasma rotation is known to excite or suppress many magnetohydrodynamic (MHD) instabilities^8,9^. Both analytical and numerical studies have shown that the sub-Alfven-speed shear flows can stabilize the tearing modes in systems comprising single or multiple periodic resonant surfaces^10–12^. When the velocity variance of the shear flows exceeds a threshold value^2^, a new variety of unstable mode, the Kelvin–Helmholtz (KH) instability^3,4,13^, appears; the growth rate of this instability is greater than tearing modes^14–20^. KH instabilities have been found to underlie various phenomena that are observed across many fields, including magnetospheric physics^5,6^, astrophysics^21,22^, dusty plasmas^23^, and fusion physics^24–26^.

Previous results have shown that in KH instabilities the magnetic field lines and flow field lines are almost parallel with each other, and that the neutral sheet and magnetic topology take a wave-like form^14–20^. In experiments, the KH instability has been studied as a possible explanation for poloidal asymmetries of density fluctuations that reverse with the plasma current direction. It has been demonstrated that these modes are localized around positions where the radial gradient of the parallel velocity takes a maximal value^27^. Strong sheared plasma flows are predicted to drive KH oscillations unstable in spherical tokamak plasmas^28^. For small shear flow thicknesses, the KH instability is excited; by contrast, for sufficiently large thicknesses, the tearing instability will be dominant^29^. The transport code predicts that the toroidal rotation in tokamak can reach the ion-sound speed^30^. At such large shear flows, magnetic KH instabilities must be considered^28^. In fusion theory and experimental research, some investigations of, e.g. KH type of instabilities in tokamak plasmas can be found^27,31–33^.

The linear growth rate of the KH modes increases with increasing strength of the shear flows in system with a single resonant surface. If the magnetic shear is sufficiently large, the tearing mode will exhibit a strong coupling to the KH instabilities and will form a new type of resistive instability driven by the KH instabilities^19,34^. For a system with two resonant surfaces, the double tearing mode (DTM) with a strong magnetic shear, the combined effect of the stabilization of the ‘in-phase’ period and the destabilization of the ‘out-of-phase’ period leads to island suppression, and even enters interlocking and saturation processes of the dual islands in the nonlinear stage^35–42^. However, when the shear flows are strong and have velocities close to or larger than the local Alfven speed, the growth of the antisymmetric resistive instability is further increased^19^. In that case, via the reconnection process on the dual resonant surfaces, DTMs can interact with each other and also couple with the KH instabilities^43^.

The nonlinear evolution of the KH instability in the presence of shear flows has been numerically calculated using a reduced MHD model^44^. This methodology is used in the case of strong shear flows and coupling with DTMs. Simulations in this regime exhibit a multi-mode interaction. In a turbulent background dominated by the KH instability, secondary magnetic islands generated by the KH instability are identified^45^; this KH-tearing mode is excited due to the generation of zonal flows^46,47^.

In the existing literature, little attention has been paid to the role of the flow profile and weak magnetic shear plasmas. In general, in the weak magnetic shear configuration, KH instabilities are more easily excited by shear flows^12^. And KH instabilities can cause magnetic reconnection^48^. In particular, in fusion plasmas with weak magnetic shear configuration, e.g., JET^49^, DIII–D^50^, and NSTX^51^, non-resonant instabilities are more relevant to ITER advanced or hybrid scenarios; this case still requires further investigation^52–56^.

In this report, we use the compressible resistive MHD model with a weak magnetic shear field and a shear flow with a hyperbolic tangent profile, focusing on weak and reversed magnetic shear plasmas. We study the dependence of the KH instability on the flow profile and the DTM-KH instability interaction mechanism. The temporal evolution of the averaged perturbed magnetic field is used to measure the growth rate of the instability. We primarily concentrate on the nonlinear phase of the instability.

This paper is organized as follows: The resistive MHD model and the numerical method are introduced in section "Model and governing equations", the results are described in section "Numerical results", and a conclusion and a discussion are presented in section "Conclusion".

## Model and governing equations

The compressibility of the plasma is considered in the simulations presented here via the use of the two-dimensional (2D) compressible MHD model. A typical sheet pinch with two layers of current flowing in opposite directions in the $z$-direction is applied; this model is discussed in Refs.^35,36^. The dimensionless compressible MHD equations can then be written as follows^36^:

1 $$\frac{\partial \rho }{{\partial t}} = - \vec{u} \cdot \nabla \rho - \rho \nabla \cdot \vec{u},$$

2 $$\frac{\partial P}{{\partial t}} = - \vec{u} \cdot \nabla P - \Gamma P\nabla \cdot \vec{u},$$

3 $$\frac{{\partial \vec{u}}}{\partial t} = - \vec{u} \cdot \nabla \vec{u} - \frac{1}{2\rho }\nabla \left( {\beta_{p} P + B^{2} } \right) + \frac{1}{\rho }\left( {\vec{B} \cdot \nabla \vec{B} + \nu \nabla^{2} \vec{u}} \right),$$

4 $$\frac{{\partial \vec{B}}}{\partial t} = - \vec{u} \cdot \nabla \vec{B} - \vec{B}\nabla \cdot \vec{u} + \vec{B} \cdot \nabla \vec{u} + \eta \nabla^{2} \vec{B},$$

where the density, $\rho$, plasma pressure, $P$, coordinates, $x$ and $y$, magnetic field, $\vec{B}$, plasma velocity, $\vec{u}$, and time, $t$, are respectively scaled by $\rho_{0}$, $P_{0}$, $L_{0}$, $B_{0}$, $u_{A} = B_{0} /\sqrt {\rho_{0} }$ and $\tau_{A} = L_{0} /u_{A}$ with $L_{0}$ being the scale length in the *x*-direction^36^. In this work, $\beta_{p}$ is the plasma beta, where $\beta_{p} = 2P_{0} /B_{0}^{2}$, and $\Gamma$ is the adiabatic index. The plasma viscosity, $\nu_{m}$, and resistivity, $\eta$, are normalized according to $\nu = \nu_{m} /\left( {u_{A} L_{0} \rho_{0} } \right)$ and $\eta = \eta_{m} /\left( {u_{A} L_{0} } \right)$, respectively. The simulation domain is limited to $- 1 \le x \le 1$ and $0 \le y \le 2$. Free and periodic boundary conditions are imposed at $x = \pm 1$ and $y = 0,2$, respectively. Equations (1)–(4) can be solved using the Runge–Kutta method with fourth-order accuracy in time. Here, a form of central-difference scheme is used to achieve second-order accuracy in space. To achieve the initial equilibrium, a magnetic field given by $B_{0y} (x) = 1 - (1 + b_{c} ){\text{sech}} (\zeta x)$, is considered, where $b_{c}$ and $\zeta$ are chosen such that the resonant surfaces are situated at $x_{s} = \pm 0.25$; we consider a weak magnetic shear given by $s = B^{\prime}_{0y} (x_{s} ) = \pi /32$; in previous studies on DTMs, $s = \pi /2$ has been used^35–39^. The initial equilibrium for $P$ is generated considering the force balance, $P = P_{0} + (B_{0}^{2} - B^{2} )/2$, and the density profile is given by $\rho = P/T$, which is based on the assumption of a constant temperature. The other parameters used in the numerical work presented here are $\Gamma = 5/3$, $\beta_{p} = 0.5$, $\eta = 5 \times 10^{ - 5}$, $\nu = 10^{ - 5}$, $\Delta x = 0.01$, $\Delta y = 0.01$, and $\Delta t = 0.002$ to satisfy the computational accuracy and Courant–Friedrichs–Lewy (CFL) numerical stability condition. In this work, we comprehensively selected an appropriate grids number and time steps size to ensure the convergence of the code.

## Numerical results

The previous results related to DTMs have shown that if the two islands move with different velocities, the combined effect of the stabilization of the ‘in-phase’ period and the destabilization of the ‘out-of-phase’ period leads to island suppression. If the strength of the shear flow is sufficiently large, the tearing mode is not excited^39^. The threshold for the onset of the KH instabilities has been given by the relation of ${\rm K} \propto \partial v_{y} /\partial x$ in Ref.^32^, where $v_{y}$ is the initial flow profile. The evolution of KH instabilities in the TEXTOR tokamak plasma have also been discussed.

**Figure 1:**
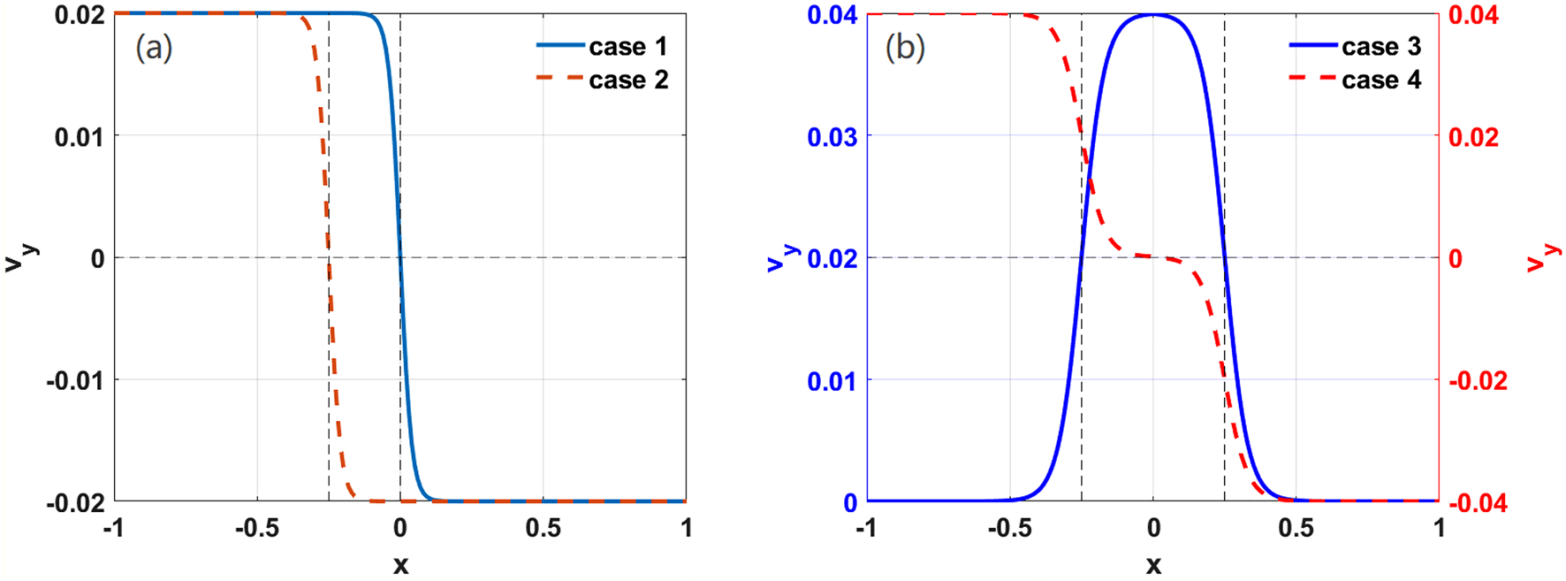
(Color online) Radial profiles of the shear flow, $v_{y}$, for cases 1, 2, 3, and 4.

To compare the results of the work undertaken here with those found in the existing literature, we consider a monotonic equilibrium shear flow^39^. The profile function in this situation can be written as $v_{y} (x) = V_{0} \tanh \left[ {\kappa \left( {x - x_{0} } \right)} \right]\hat{y}$, where $V_{0}$ and $\kappa$ are the velocity and shear factors of the flow, respectively. Previous studies have shown that the tearing mode is more easily formed on rational surfaces^57^. If shear flow is absent under the parameter conditions set here, DTM will form at the two resonant surfaces, as can be seen in the 2D structure of the total magnetic flux later. In order to clarify the effect of rational surface on the KH instabilities, in Fig. 1a, we consider two cases: In case 1, the flow shear on the two resonant surfaces is taken to be zero; a strong flow shear at *x* = 0 is considered to study the effect of the flow shear on the KH instability in weak and reversed magnetic shear plasmas. In case 2, which is considered in order to offer a comparison with case 1, the local strong flow shear is imposed at left resonant surface.

In the case of the DTMs, the shear or relative velocity ($\tilde{v} \equiv \left| {v_{s1} - v_{s2} } \right|$) of the two resonant surfaces; plays an important role in suppression of the mode. To further identify whether $\tilde{v}$ is the dominant factor in the excitation of KH instabilities, we introduce cases 3 and 4 in Fig. 1b. In case 3, the velocity and shear strength on both the resonant surfaces are same, and, thus, $\tilde{v}$ is zero. In case 4, the shear strength on the two resonant surfaces is equal and simultaneously moves backwards at a speed of 0.02.

**Figure 2:**
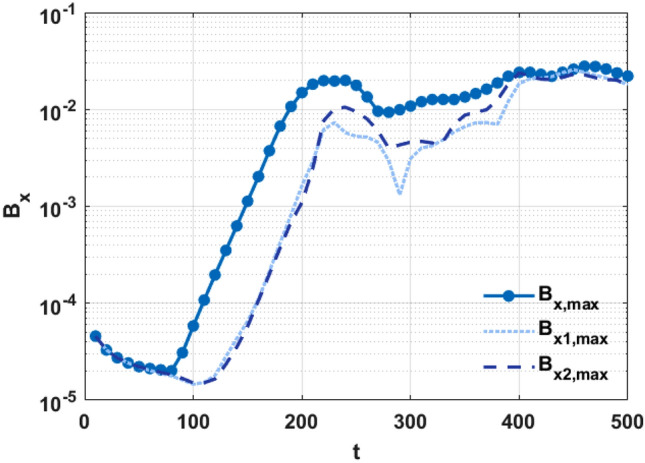
(Color online) Temporal evolution of the magnetic perturbation, $B_{x}$, with shear flow (case 1). $B_{x,\max }$ represents the maximum value of $B_{x}$. The parameters $B_{x1,\max }$ and $B_{x2,\max }$ represent the maximum value of $B_{x}$ on the left and right resonant surface, respectively.

To show the nonlinear evolution of the instability, the maximum value of perturbed magnetic field $B_{x}$ as a function of time is shown in Fig. 2. It can be seen that the KH instability is driven by the shear flow, despite the flow shear being weak on the resonant surfaces. This result is significantly different from the results obtained in the case of a single resonant surface; in the single resonant case, a strong flow shear is required to drive KH instabilities^10,24–26^. In case 1, the linear growth rates of $B_{x}$ on the two resonant surfaces are almost equal. Moreover, the instability with the largest value of $B_{x}$ on the linear phase is not necessarily localized on the two resonant surfaces for $B_{x,\max } > B_{x1,\max } ,B_{x2,\max }$; the symmetric mode structures generated on dual resonant surfaces are obtained for $B_{x1,\max } \approx B_{x2,\max }$. In the nonlinear stage, the value of $B_{x}$ on the two resonant surfaces gradually increases to $B_{x,\max } \approx B_{x1,\max } \approx B_{x2,\max }$ especially after $t \sim 400$.

**Figure 3:**
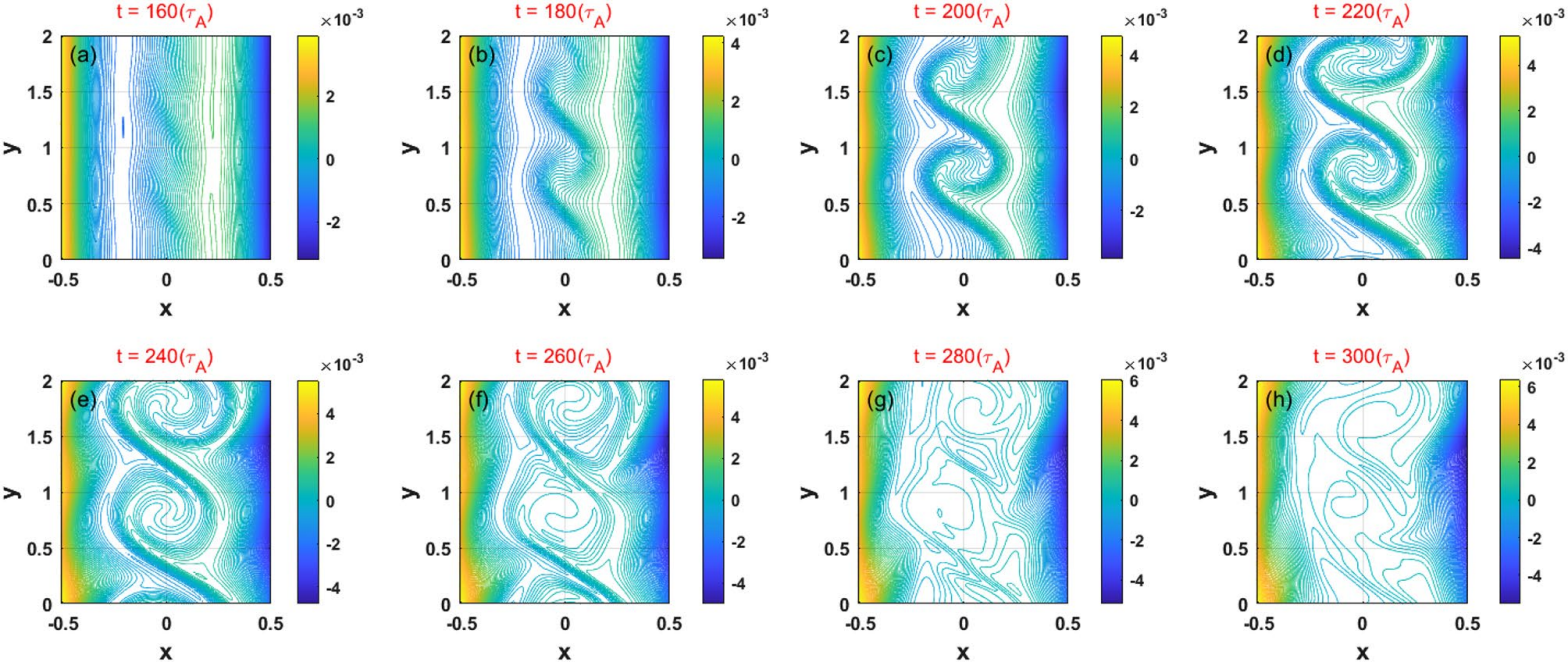
(Color online) The 2D structure of the total magnetic flux in the presence of a KH instability for various values of $t$ for case 1.

To show the mode structure, in Fig. 3, we plot 2D contours of the magnetic flux, $\psi$, with $\vec{B} = \nabla \psi \times \hat{z}$. The magnetic field lines are curled significantly between two resonant surfaces due to the strong flow shear. This suggests that the mode may be a non–resonant mode in the absence of a resonant surface at $x = 0$. At the end of linear growth regime ($t\sim 220$), however, the resonant tearing–like instability can also be driven by the KH–induced plasma flows on the resonant surfaces; this is similar to a process of forced magnetic field reconnection^45,54,55^. This situation is distinct from strong shear case^18,19^, as in this case, both KH and tearing instabilities are not coupling, i.e., the KH instability always plays a dominant role in the evolution of the nonlinear magnetic field. After the system evolved for a long time, the MHD nonlinearity leads to the generation similar to those observed in turbulence structures.

**Figure 4:**
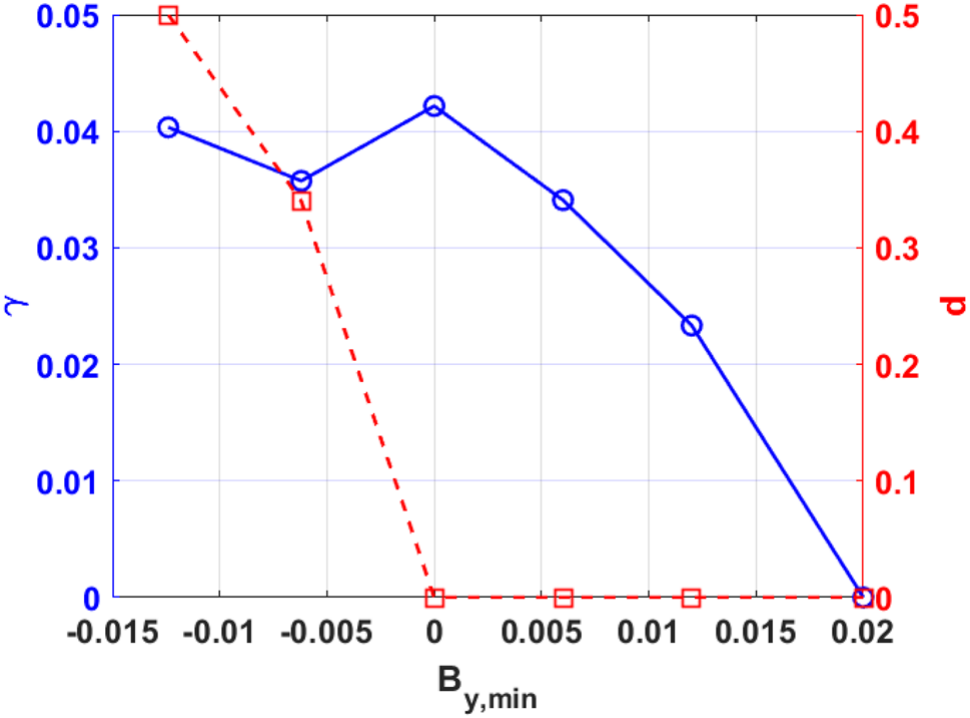
(Color online) The growth rate (solid curve, $\gamma = d\ln B_{x} /dt$) and the distance of between the two resonant surfaces (dashed curve, $d = 2\left| {x_{s} } \right|$) as a function of the minimum value of $B_{y}$.

In cases involving reversed magnetic shear, the distance between the two resonant surfaces has an important effect on the resistive MHD instabilities^8,36^. In Fig. 4, we increase the initial values of $B_{y}$ in order to study how the mode growth rate depends on the distance between the two resonant surfaces, where the growth rate of KH modes $\gamma = d\ln B_{x} /dt$ and the distance of between the two resonant surfaces $d = 2\left| {x_{s} } \right|$. It is interesting to note that as the distance decreases to zero, the growth rate does not change significantly. This result suggests that the KH mode coupling between the two resonant surfaces is very weak in this case. It also should be noted that not only the distance but also the magnetic shear on the resonant surfaces becomes small with increasing values of $B_{y}$. Situations with weak magnetic shear plasmas are more likely to lead to the excitation of the KH mode than those with strong magnetic shear plasmas^12^. This suggests that in weak magnetic shear regimes, the excitation of the KH mode does not depend on the position of resonant surfaces. Furthermore, once the mode becomes a non–resonant mode as $B_{y,\min } > 0$^52^, the strong stabilizing effect of field line bending on the KH mode dominates the mode linear growth with the position of the mode being far away from the resonant surfaces. Yao et al. studied the effect of distance between two resonant surfaces on DTMs using gyrokinetic code^59^. Their research found that as the separation of the rational surfaces was increased, the growth rates of DTMs were enhanced and the DTM system tended to decouple into a system of two single-tearing modes. Interestingly, the distance between two rational surfaces has different mechanisms of influence on the instability of DTMs and KH modes. The relevant mechanisms need to be studied in detail.

**Figure 5:**
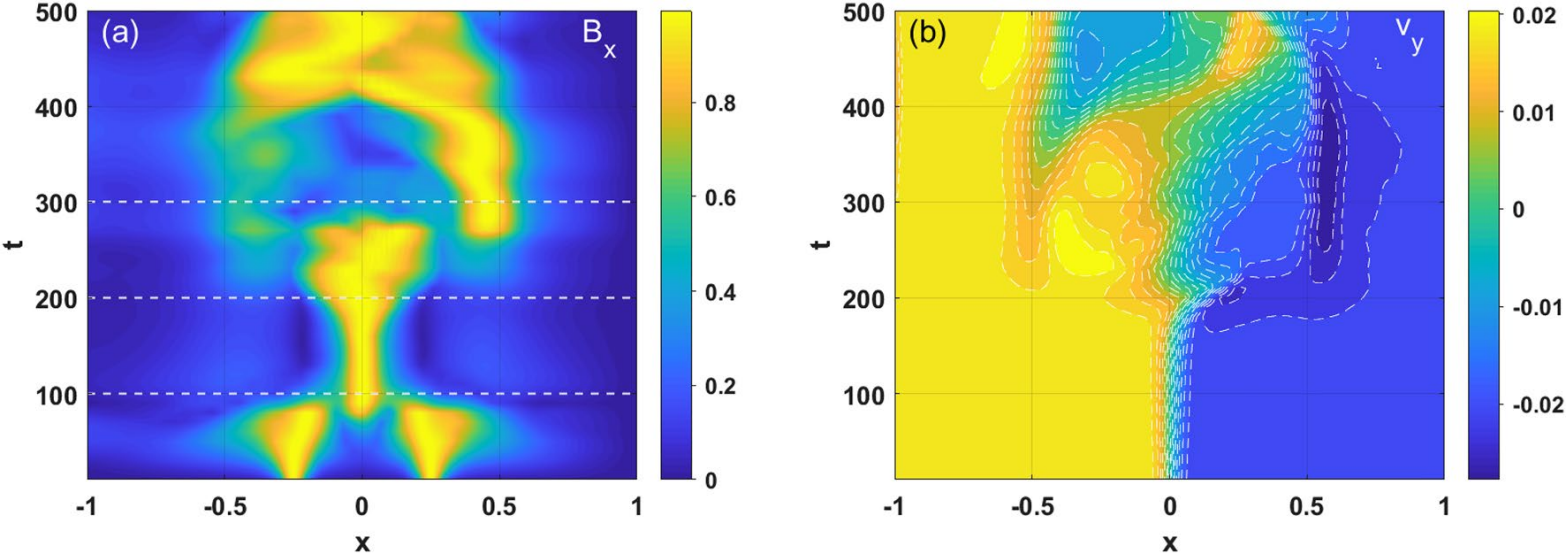
(Color online) Temporal and spatial evolution of (**a**) the magnetic perturbation and (**b**) shear flow ($v_{y}$) for case 1.

The temporal evolution of the magnetic perturbation profile in *x*-direction is depicted in Fig. 5a. Here, in order to compare the mode structure at different times, $B_{x}$ is normalized by $B_{x,\max }$ (the same applies below). It can be seen that the initial perturbation is localized on the two resonant surfaces (i.e., $x_{s} = \pm 0.25$) and damped over time due to the relaxation process ($t < 100$), as shown in Fig. 2. In this case, the tearing mode is stable due to the weak magnetic shear regimes. The KH instability exhibits a linear growth regime with a more localized spatial structure than that observed in the case DTMs for $100 < t < 200$. However, when the mode enters the nonlinear regime, the spatial structure of the KH mode broadens, and the resistive magnetic reconnection is then driven by KH instabilities that have a vortex structure of island–like shapes on the resonant surfaces. Due to the strong coupling of KH modes in the presence of resonant surfaces, the equilibrium flow profile is modified, as shown in Fig. 5b; in Fig. 5b, $y = 0$ is fixed (the same applies below). It is notable that in the early nonlinear growth stage, the KH mode produces an additional flow in the same direction as the initial flow near the resonant surface.

**Figure 6:**
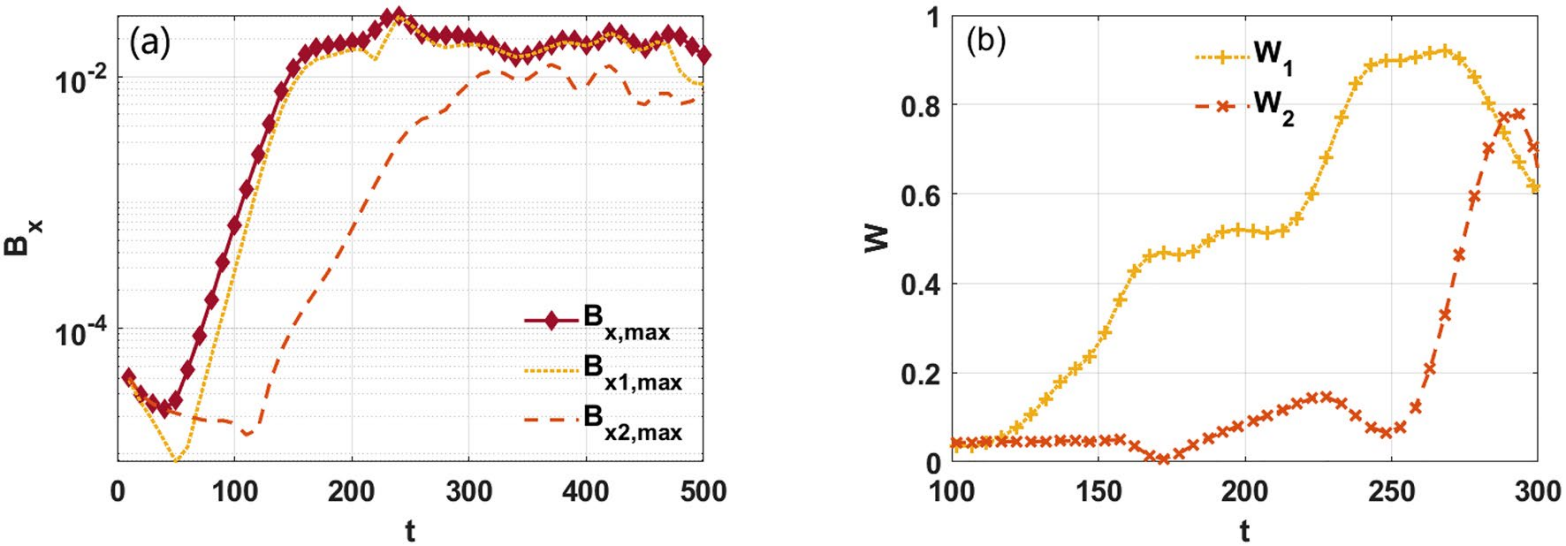
(Color online) (**a**) Temporal evolution of the magnetic perturbation ($B_{x}$) for case 2. (**b**) The width of the left ($W_{1}$) and right ($W_{2}$) islands as a function of time.

To show the nonlinear evolution of the instability, the maximum value of perturbed magnetic field $B_{x}$ as a function of time is shown in Fig. 6a. In case 1, the flow profile is symmetry at point $x = 0$. The flow profile is usually variable in astrophysical or tokamak plasmas^1–7,21^. In order to further elucidate the effect of the flow profile on the KH modes in the weak and reversed magnetic shear configuration, we study a second case (case 2) in which we translate the position of the flow shear to $x = - 0.25$. Comparing the two cases, we see that the KH mode is more likely to be excited in case 2 due to the strong flow shear on the resonant surface. The asymmetric magnetic perturbation forms on both the resonant surfaces as $B_{x,\max } \approx B_{x1,\max } > B_{x2,\max }$. The saturated amplitude of $B_{x1,\max }$ is also larger than 2 times that of $B_{x2,\max }$. It is expected that, similar to the case of a single resonant surface, the islands induced by the KH instabilities on left resonant surface can induce an interaction between the tearing mode and the KH mode that then drive each other^14–19^. We see that the island structure begins to grow when the KH mode becomes sufficiently strong, as shown in Fig. 6b. $W$ shows the width of the islands. Nevertheless, early within the nonlinear stage, the right island grows very slowly until an inward flow form at $t > 250$^21,22,35^.

**Figure 7:**
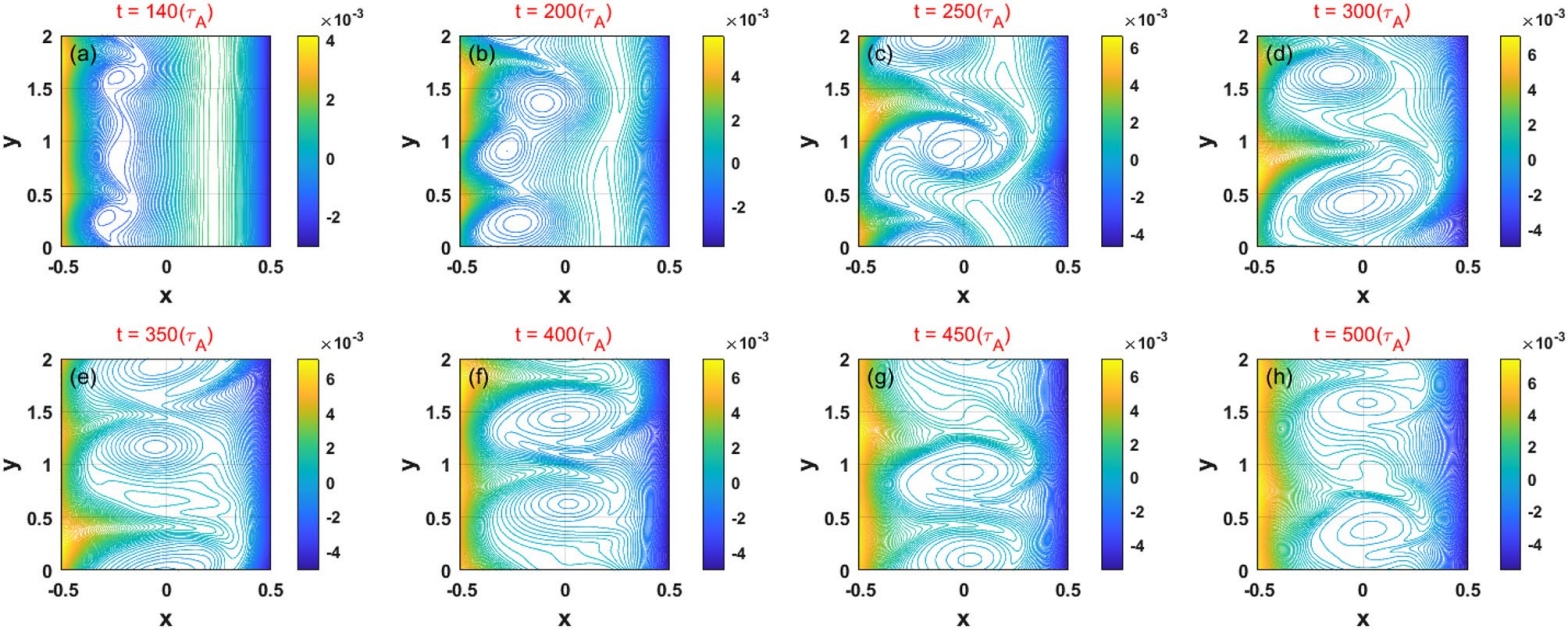
(Color online) Temporal evolution of the 2D features of the total magnetic flux of the KH instability for case 2.

To capture the nonlinear evolution of case 2, we obtained 2D contours of the magnetic flux in this case, as shown in Fig. 7. It can be seen that the position of the flow shear plays an important role in the formation of the KH and tearing modes. A high-mode number harmonic of the island on the left resonant surface is induced by wavy magnetic and flow field; in this case, the islands grow quickly and bring about the coalescence of the mode harmonics. It is notable that unlike in previous simulations of KH-tearing coupled modes^14–19^, we find that the KH mode always dominates in the dynamics over the tearing mode; this observation represents an important phenomenon in the weak and reversed magnetic shear configuration. A rotating island generated on the right resonant surface keeps interlocking with the KH modes in the nonlinear stage ($200 < t < 350$); these modes eventually overlap with each other. Furthermore, for $t > 400$, the KH instability saturates at a high number level with the two large size islands that exist between the two resonant surfaces. The initial magnetic topology is deformed and two eddy–like structures are generated; these eddy-like structures may result in a further enhancement of radial plasma transport. Therefore, in order to maintain a stable configuration, sheared plasma flows below the critical level are required in weak and reversed magnetic shear configuration.

**Figure 8:**
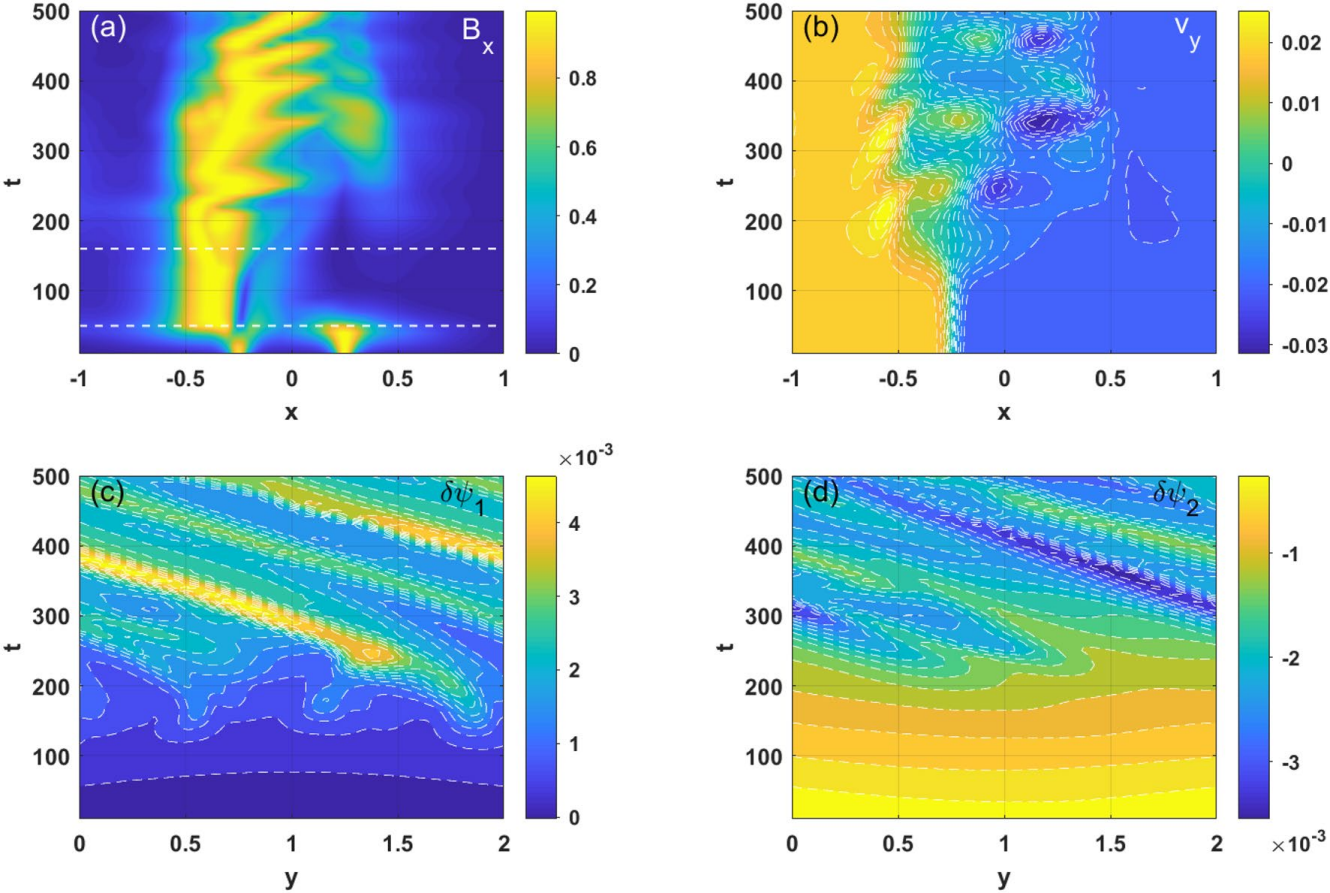
(Color online) Temporal and spatial evolution of (**a**) the magnetic perturbation, (**b**) the shear flow, and (c and d) the perturbations of the magnetic flux on the left and right resonant surface ($\delta \psi_{1}$ and $\delta \psi_{2}$), respectively, for case 2.

From Fig. 8a, it can be seen that in case 2 the KH mode becomes dominant earlier than it does in case 1. An asymmetric perturbed magnetic structure is driven only on the left resonant surface in the linear stage, which is distinct from the results obtained related to case 1 (as shown in Fig. 5a). In the nonlinear stage of the KH mode, we see interlocking as well as coupling between the two resonant surfaces for $t > 300$; meanwhile, it can be seen that the KH modes are moving toward each other in the *x*-direction. Figure 8b shows that the flow profile remains constant in the early linear stage ($t < 100$). When the KH–induced islands start to grow, the flow profile first flattens near the left resonant surface. The relative velocity of the two resonant surfaces then decreases due to interlocking of the two KH modes. Once the interactions between the surfaces become sufficiently strong, the zonal flow structure will alternatively appear and move to the region close to $x = 0$. A possible reason for this phenomenon is that the zonal flow arises due to the magnetic reconnection process, which also causes two opposite resonant surfaces to attract each other, as is the case with DTMs^35^. Figure 8c,d show the perturbed magnetic flux on the left and right resonant surfaces, respectively. It can be seen that the high mode number harmonics are excited due to the large flow shear on the resonant surface at the linear and early nonlinear states of the KH modes. However, they merge rapidly with each other for $t > 200$. The coupled KH modes rotate together and enter a long term nonlinear dynamic process. A different behavior can be observed on the right resonant surface due to the asymmetry of the flow profile considered in the simulation.

**Figure 9:**
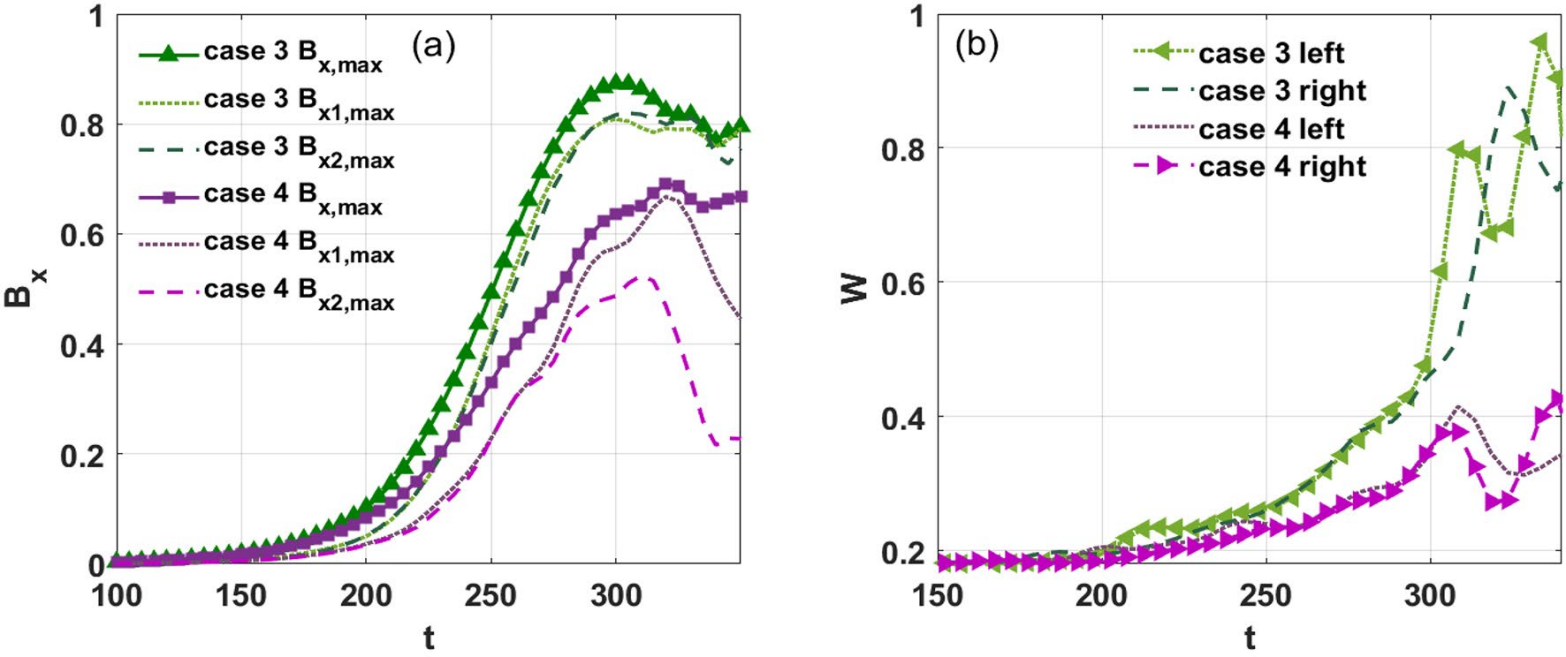
(Color online) (**a**) Temporal evolution of the maximum value of magnetic perturbation with shear flow in cases 3 and 4. (**b**) The width of the islands as a function of time.

To show the nonlinear evolution of the instability, the maximum value of perturbed magnetic field $B_{x}$ as a function of time is shown in Fig. 9a. When the absolute value of velocity and shear are the same, we see that $B_{x,\max }$, $B_{x1,\max }$, and $B_{x2,\max }$ in case 3 are greater than those in case 4. In the interval $200 < t < 300$, the relative rotation of the two resonant surfaces suppresses the KH modes, as shown in Fig. 9a. This indicates that a finite value of $\tilde{v}$ is necessary to reduce the strength of the KH modes. We conclude that the magnetic island width is stable for $t < 150$, and it grows at a faster rate in case 3 than it does in case 4 when the driving of the KH modes is sufficiently strong. Since the combined effect of the stabilization of the ‘in-phase’ period and the destabilization of the ‘out-of-phase’ period leads to island suppression^39^, the island width in case 4 is also seen to saturate at a lower value than in case 3. Moreover, we find that the two islands of both sides are almost symmetric during the growth stage.

**Figure 10:**
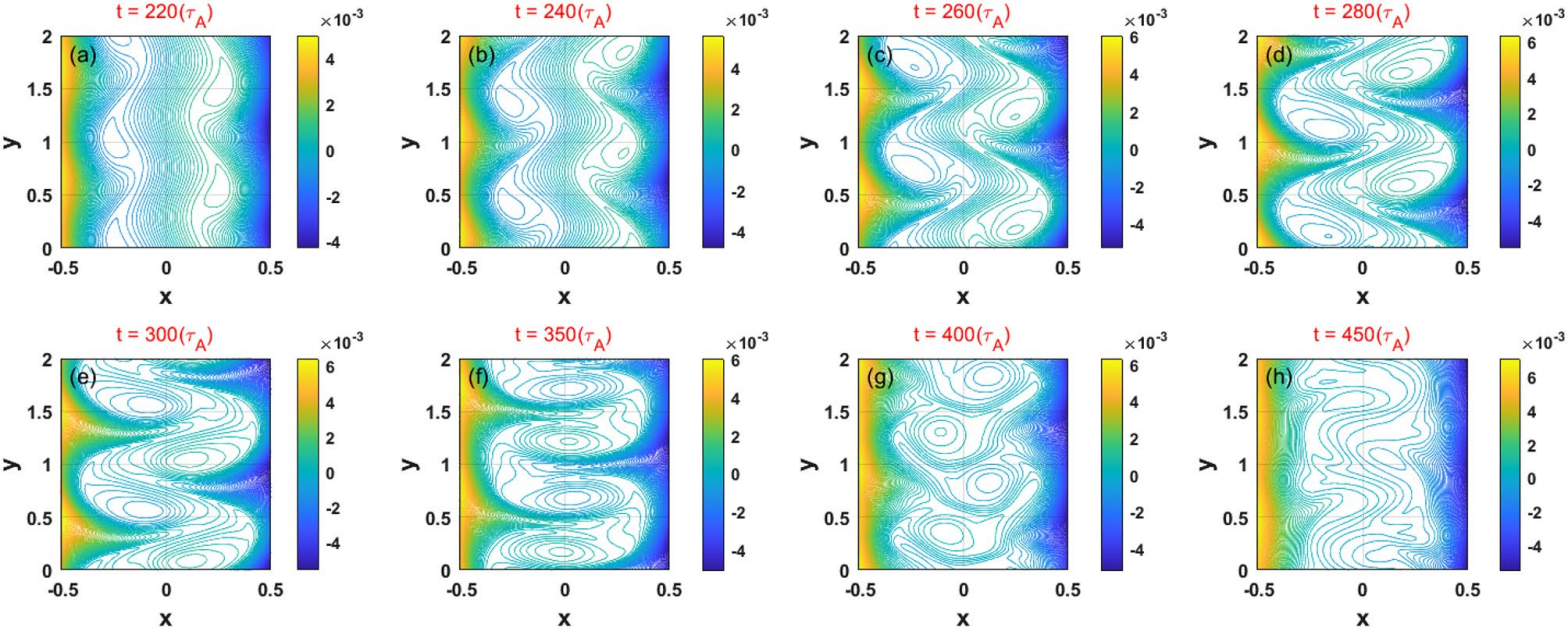
(Color online) Temporal evolution of the 2D total magnetic flux related to the KH instability in case 3.

The nonlinear dynamics of the KH modes on the two resonant surfaces in case 3 are shown in Fig. 10. Here, the wave number is dominated by $k_{y} \sim 2$, which is larger than the $k_{y} \sim 1$ observed frequently in DTMs considering simulation parameters in the case of strong magnetic shear^39,41^. It is found that the KH–induced modes can also drive each other; we note that they also rotate in the $y$ direction but do not present any relative motion, which results in an asymmetric forced driven magnetic reconnection configuration to be well remained. In the nonlinear saturated stage, the field lines between the resonant surfaces are reconnected, and the two modes overlap in a similar manner to that observed in standard DTMs^35^. However, we note that the secondary islands usually observed in the simulation of DTMs are not obvious in this weak and reversed magnetic shear case.

**Figure 11:**
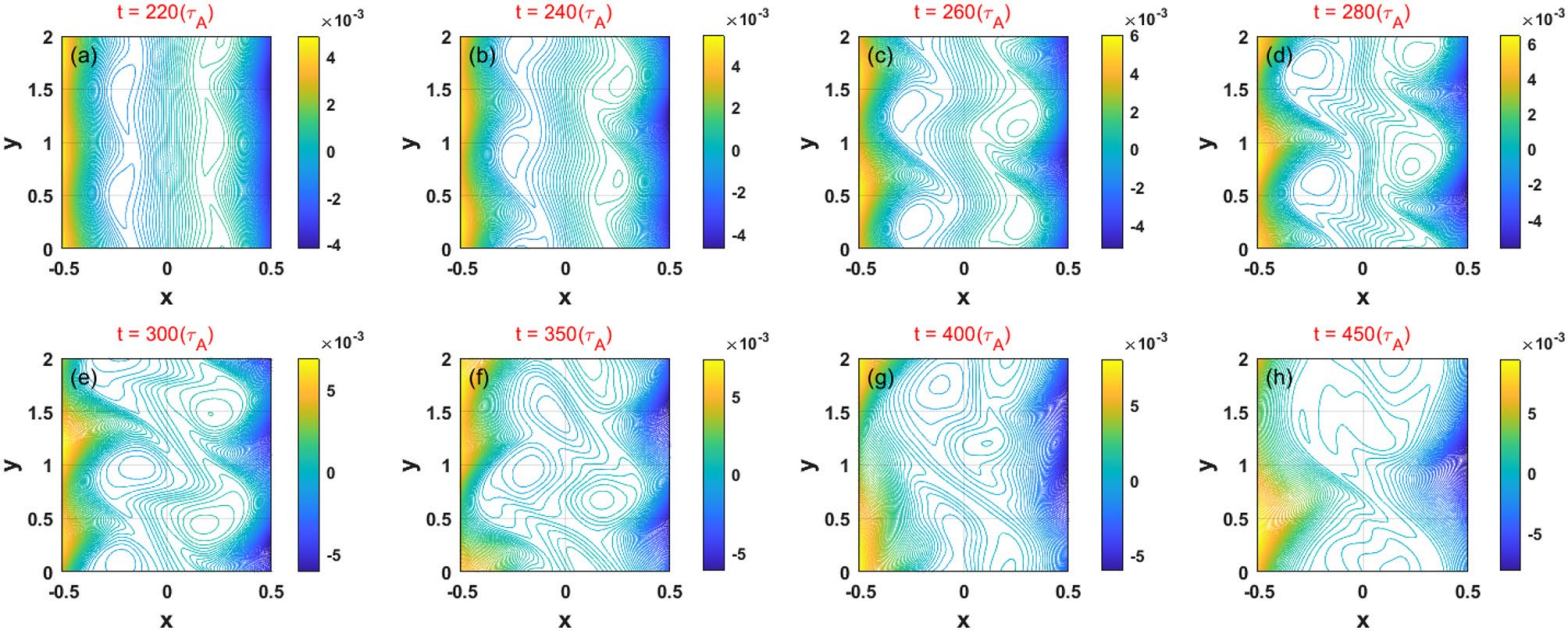
(Color online) Temporal evolution of the 2D total magnetic flux related to the KH instability in case 4.

The interlocking process of the two KH instabilities is shown in Fig. 11. Unlike the interlocked DTMs, we see that the deformation of the KH modes is a result of the effect of both flow shear and mode coupling. In the nonlinear stage, the interaction of two KH modes induces the destruction of the islands and subsequently twists then together between the two resonant surfaces. Thus, the model that includes the effects of compressibility is more suitable to the study of interlocking KH modes in the weak magnetic shear configuration compared with the reduced MHD model.

## Conclusion

In this report, we have simulated the nonlinear interaction between DTMs and KH instabilities with different shear flow profiles using the compressible MHD model. It was found that the KH instability can be driven by shear flow with the coupling of two KH modes in a weak and reversed magnetic shear configuration. The excitation of the KH mode does not have a dependence on the positions of the resonant surfaces in the system with two resonant surfaces. In particular, when the mode becomes a non–resonant mode, the strong stabilizing effect of field line bending on the KH instability dominates the linear growth. The nonlinear dynamics of the coupling between the KH mode and the tearing mode on the two resonant surfaces were discussed in detail. In these cases, the KH mode is seen to dominate the instability dynamics, which suggests that weak magnetic shear regimes are critical to the formation of the KH structure. This result is also agreement with the theory of Ref.^12^.

Furthermore, it was found that the relative rotation of the two resonant surfaces has a significant suppression effect on the KH modes. In the case of a symmetric flow, an asymmetric forced magnetic reconnection configuration is maintained and leads to locking of double KH modes, but the secondary islands usually observed in simulation of DTMs are not obvious in the weak and reversed magnetic shear cases. Unlike in the case of interlocked DTMs, we also found that the wavy magnetic field of KH modes is a result of the synergistic effects of flow shear and mode coupling. In the nonlinear stage, the interaction of the two KH modes induces the destruction of the islands and subsequently twists them together between the two resonant surfaces. The study of the interlocking process of the two KH instabilities may be useful to understand the mechanism of nonlinear reconnection in weak and reversed magnetic shear plasmas. However, plasma compressibility, guild field and relativistic effects^58^ could also play an important role on understanding of interlocking of KH modes in the weak magnetic shear configuration, should be considered in more detail.

## Data Availability

All data generated or analyzed during this study are included in this published article.
